# (1*E*,2*E*)-1,2-Bis[1-(3-nitro­phen­yl)ethyl­idene]hydrazine

**DOI:** 10.1107/S1600536812004722

**Published:** 2012-02-10

**Authors:** Safra Izuani Jama Asik, Hoong-Kun Fun, Ibrahim Abdul Razak, Patcharaporn Jansrisewangwong, Suchada Chantraproma

**Affiliations:** a X-ray Crystallography Unit, School of Physics, Universiti Sains Malaysia, 11800 USM, Penang, Malaysia; b Crystal Materials Research Unit, Department of Chemistry, Faculty of Science, Prince of Songkla University, Hat-Yai, Songkhla 90112, Thailand

## Abstract

The asymmetric unit of the title compound, C_16_H_14_N_4_O_4_, contains one half-mol­ecule of (nitro­phen­yl)ethanimine and the complete mol­ecule is generated by a crystallographic inversion centre. The mol­ecule has an *E* conformation with respect to each C=N double bond. The central C=N—N=C plane is twisted from the benzene rings with a dihedral angle of 24.76 (11)°. In the crystal, C—H⋯O inter­actions link the molecules to form sheets that lie parallel to (10-4).

## Related literature

For the biological acivity of hydrazones, see: Khanmohammadi *et al.* (2008 ▶); Luboch *et al.* (2009 ▶). For related structures, see: Chantrapromma *et al.* (2011 ▶); Fun, Jansrisewangwong *et al.* (2011 ▶); Fun, Nilwanna *et al.* (2011 ▶); Jansrisewangwong *et al.* (2010 ▶); Nilwanna *et al.* (2011 ▶). For bond-length data, see: Allen *et al.* (1987 ▶).
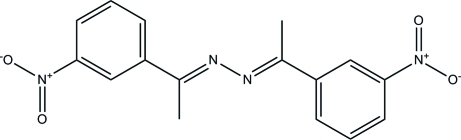


## Experimental

### 

#### Crystal data

C_16_H_14_N_4_O_4_
*M* *_r_* = 326.31
Monoclinic, 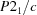

*a* = 3.9296 (3) Å
*b* = 7.4448 (5) Å
*c* = 26.3979 (19) Å
β = 94.022 (1)°
*V* = 770.37 (10) Å^3^
*Z* = 2
Mo *K*α radiation
μ = 0.10 mm^−1^
*T* = 296 K
0.34 × 0.17 × 0.10 mm

#### Data collection

Bruker APEX DUO CCD area-detector diffractometer
Absorption correction: multi-scan (*SADABS*; Bruker, 2009 ▶) *T*_min_ = 0.966, *T*_max_ = 0.990
15392 measured reflections
2254 independent reflections
1686 reflections with *I* > 2σ(*I*)
*R*_int_ = 0.028

#### Refinement

*R*[*F* ^2^ > 2σ(*F* ^2^)] = 0.047
*wR*(*F* ^2^) = 0.146
*S* = 1.06
2254 reflections
110 parameters
H-atom parameters constrained
Δρ_max_ = 0.21 e Å^−3^
Δρ_min_ = −0.18 e Å^−3^

### 

Data collection: *APEX2* (Bruker, 2009 ▶); cell refinement: *SAINT* (Bruker, 2009 ▶); data reduction: *SAINT*; program(s) used to solve structure: *SHELXTL* (Sheldrick, 2008 ▶); program(s) used to refine structure: *SHELXTL*; molecular graphics: *SHELXTL*; software used to prepare material for publication: *SHELXTL* and *PLATON* (Spek, 2009 ▶).

## Supplementary Material

Crystal structure: contains datablock(s) global, I. DOI: 10.1107/S1600536812004722/is5067sup1.cif

Structure factors: contains datablock(s) I. DOI: 10.1107/S1600536812004722/is5067Isup2.hkl

Supplementary material file. DOI: 10.1107/S1600536812004722/is5067Isup3.cml

Additional supplementary materials:  crystallographic information; 3D view; checkCIF report

## Figures and Tables

**Table 1:** Hydrogen-bond geometry (Å, °)

*D*—H⋯*A*	*D*—H	H⋯*A*	*D*⋯*A*	*D*—H⋯*A*
C3—H3*A*⋯O1^i^	0.93	2.57	3.239 (2)	129

Symmetry code: (i) 
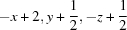
.

**Fig. 1.**
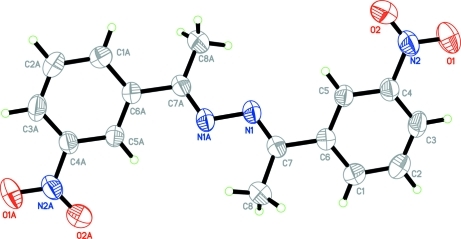
The structure of the title compound, showing 50% probability displacement ellipsoids. H atoms are shown as spheres of arbitrary radius.

**Fig. 2.**
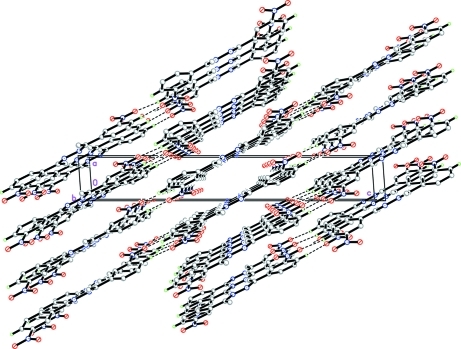
The crystal packing, viewed along the b-axis, showing the molecules linked into two-dimensional layers parallel to the (104) plane. H atoms that not involved in hydrogen bonding (dashed lines) are omitted for clarity.

## supplementary crystallographic information

### Comment

Hydrazones have been intensively investigated mostly because of their potential applications as antibacterial and antifungal drugs (Khanmohammadi *et al.*, 2008) and as fluorescent chemosensors for metal ions (Luboch *et al.*, 2009). Owing to our medicinal chemistry research on hydrazones, we previously reported the synthesis and crystal structures of some hydrazone derivatives (Chantrapromma *et al.*, 2011; Fun, Jansrisewangwong *et al.*, 2011; Fun, Nilwanna *et al.*, 2011; Jansrisewangwong *et al.*, 2010; Nilwanna *et al.*, 2011). The title compound was synthesized to study for antibacterial activity and fluorescence properties in order to get more detail on the structural activity relationship through comparing with other closely related compounds.

The asymmetric unit of (I) (Fig. 1), contains one half-molecule of (nitrophenyl)ethanimine and the complete molecule is generated by a crystallographic inversion centre (-x,-y + 1,-z). The molecule is in an *E* configuration with respect to C7═N1 double bond [1.2803 (17) Å] with the torsion angle N1A—N1—C7—C1 = 179.92 (10)°. The methyl groups are twisted from the planes of benzene (C1–C6 and C1A–C6A) rings and their orientations can be indicated by the torsion angles (C1—C6—C7—C8 and C1A—C6A—C7A—C8A) = 24.91 (19)°. The bond length are within the normal range (Allen *et al.*, 1987) and are comparable with the related structures (Chantrapromma *et al.*, 2011; Fun, Jansrisewangwong *et al.*, 2011; Fun, Nilwanna *et al.*, 2011; Jansrisewangwong *et al.*, 2010; Nilwanna *et al.*, 2011). In the crystal structure (Fig. 2), the C3—H3A···O1 interaction links the molecules into two-dimensional layers parallel to the (1 0 4) plane.

### Experimental

The title compound was synthesized by mixing a solution (1:2 molar ratio) of hydrazine hydrate (0.10 ml, 2 mmol) and 3-nitroacetophenone (0.66 g, 4 mmol) in ethanol (20 ml). The resulting solution was refluxed for 4 h, yielding the yellow crystalline solid. The resultant solid was filtered off and washed with methanol. Yellow block-shaped single crystals of the title compound suitable for X-ray structure determination were recrystalized from acetone by slow evaporation of the solvent at room temperature over several days (m.p. 469–471 K).

### Refinement

All the H atoms were positioned geometrically and refined using a riding model with C—H = 0.93 and 0.96 Å. The *U*_iso_ values were constrained to be 1.5*U*_eq_ of the carrier atom for methyl H atoms and 1.2*U*_eq_ for the remaining H atoms. A rotating group model was used for the methyl groups.

### Crystal data

**Table:**

C_16_H_14_N_4_O_4_	*F*(000) = 340
*M**_r_* = 326.31	*D*_x_ = 1.407 Mg m^−^^3^
Monoclinic, *P*2_1_/*c*	Mo *K*α radiation, λ = 0.71073 Å
Hall symbol: -P 2ybc	Cell parameters from 4585 reflections
*a* = 3.9296 (3) Å	θ = 2.8–28.3°
*b* = 7.4448 (5) Å	µ = 0.10 mm^−^^1^
*c* = 26.3979 (19) Å	*T* = 296 K
β = 94.022 (1)°	Block, yellow
*V* = 770.37 (10) Å^3^	0.34 × 0.17 × 0.10 mm
*Z* = 2	

### Data collection

**Table:**

Bruker APEX DUO CCD area-detector diffractometer	2254 independent reflections
Radiation source: fine-focus sealed tube	1686 reflections with *I* > 2σ(*I*)
Graphite monochromator	*R*_int_ = 0.028
φ and ω scans	θ_max_ = 30.1°, θ_min_ = 1.6°
Absorption correction: multi-scan (*SADABS*; Bruker, 2009)	*h* = −5→5
*T*_min_ = 0.966, *T*_max_ = 0.990	*k* = −10→10
15392 measured reflections	*l* = −37→37

### Refinement

**Table:**

Refinement on *F*^2^	Primary atom site location: structure-invariant direct methods
Least-squares matrix: full	Secondary atom site location: difference Fourier map
*R*[*F*^2^ > 2σ(*F*^2^)] = 0.047	Hydrogen site location: inferred from neighbouring sites
*wR*(*F*^2^) = 0.146	H-atom parameters constrained
*S* = 1.06	*w* = 1/[σ^2^(*F*_o_^2^) + (0.069*P*)^2^ + 0.1466*P*] where *P* = (*F*_o_^2^ + 2*F*_c_^2^)/3
2254 reflections	(Δ/σ)_max_ < 0.001
110 parameters	Δρ_max_ = 0.21 e Å^−^^3^
0 restraints	Δρ_min_ = −0.18 e Å^−^^3^

### Special details

**Table:**

Geometry. All esds (except the esd in the dihedral angle between two l.s. planes) are estimated using the full covariance matrix. The cell esds are taken into account individually in the estimation of esds in distances, angles and torsion angles; correlations between esds in cell parameters are only used when they are defined by crystal symmetry. An approximate (isotropic) treatment of cell esds is used for estimating esds involving l.s. planes.
Refinement. Refinement of F^2^ against ALL reflections. The weighted R-factor wR and goodness of fit S are based on F^2^, conventional R-factors R are based on F, with F set to zero for negative F^2^. The threshold expression of F^2^ > 2sigma(F^2^) is used only for calculating R-factors(gt) etc. and is not relevant to the choice of reflections for refinement. R-factors based on F^2^ are statistically about twice as large as those based on F, and R- factors based on ALL data will be even larger.

### Fractional atomic coordinates and isotropic or equivalent isotropic displacement parameters (Å^2^)

**Table:**

	*x*	*y*	*z*	*U*_iso_*/*U*_eq_	
O1	0.9642 (5)	0.1248 (2)	0.20045 (5)	0.1005 (6)	
O2	0.8102 (4)	0.06399 (18)	0.12349 (5)	0.0867 (5)	
N1	0.0796 (3)	0.47453 (16)	0.02358 (4)	0.0461 (3)	
N2	0.8220 (3)	0.16404 (18)	0.15972 (5)	0.0572 (3)	
C1	0.3995 (4)	0.6788 (2)	0.14058 (5)	0.0507 (3)	
H1A	0.3117	0.7942	0.1362	0.061*	
C2	0.5585 (4)	0.6293 (2)	0.18705 (6)	0.0583 (4)	
H2A	0.5726	0.7113	0.2137	0.070*	
C3	0.6956 (4)	0.4607 (2)	0.19421 (5)	0.0525 (4)	
H3A	0.8039	0.4272	0.2252	0.063*	
C4	0.6669 (3)	0.34261 (18)	0.15379 (5)	0.0429 (3)	
C5	0.5072 (3)	0.38640 (17)	0.10740 (4)	0.0400 (3)	
H5A	0.4914	0.3029	0.0811	0.048*	
C6	0.3697 (3)	0.55758 (17)	0.10032 (4)	0.0390 (3)	
C7	0.1991 (3)	0.60690 (18)	0.05020 (5)	0.0399 (3)	
C8	0.1829 (5)	0.7991 (2)	0.03461 (6)	0.0643 (4)	
H8A	0.1410	0.8068	−0.0016	0.096*	
H8B	0.3956	0.8567	0.0447	0.096*	
H8C	0.0016	0.8578	0.0507	0.096*	

### Atomic displacement parameters (Å^2^)

**Table:**

	*U*^11^	*U*^22^	*U*^33^	*U*^12^	*U*^13^	*U*^23^
O1	0.1420 (14)	0.0814 (10)	0.0708 (9)	0.0246 (9)	−0.0438 (9)	0.0216 (7)
O2	0.1242 (12)	0.0551 (7)	0.0763 (9)	0.0281 (7)	−0.0242 (8)	−0.0045 (6)
N1	0.0550 (6)	0.0460 (6)	0.0357 (5)	0.0002 (5)	−0.0080 (4)	0.0071 (4)
N2	0.0638 (8)	0.0518 (7)	0.0535 (7)	0.0030 (6)	−0.0133 (6)	0.0126 (6)
C1	0.0561 (8)	0.0465 (7)	0.0488 (8)	0.0046 (6)	−0.0020 (6)	−0.0061 (6)
C2	0.0696 (10)	0.0617 (9)	0.0424 (7)	−0.0022 (7)	−0.0053 (6)	−0.0130 (6)
C3	0.0576 (8)	0.0632 (9)	0.0350 (6)	−0.0071 (7)	−0.0088 (5)	0.0015 (6)
C4	0.0440 (6)	0.0450 (7)	0.0388 (6)	−0.0030 (5)	−0.0044 (5)	0.0058 (5)
C5	0.0430 (6)	0.0426 (7)	0.0338 (6)	−0.0013 (5)	−0.0024 (4)	0.0006 (5)
C6	0.0383 (6)	0.0425 (6)	0.0357 (6)	0.0002 (5)	−0.0007 (4)	0.0015 (5)
C7	0.0391 (6)	0.0426 (7)	0.0376 (6)	0.0045 (5)	0.0003 (4)	0.0042 (5)
C8	0.0847 (11)	0.0450 (8)	0.0600 (9)	0.0046 (8)	−0.0171 (8)	0.0064 (7)

### Geometric parameters (Å, º)

**Table:**

O1—N2	1.2121 (16)	C3—C4	1.3808 (19)
O2—N2	1.2105 (19)	C3—H3A	0.9300
N1—C7	1.2803 (17)	C4—C5	1.3756 (17)
N1—N1^i^	1.406 (2)	C5—C6	1.3916 (18)
N2—C4	1.4663 (19)	C5—H5A	0.9300
C1—C2	1.387 (2)	C6—C7	1.4867 (17)
C1—C6	1.3930 (19)	C7—C8	1.489 (2)
C1—H1A	0.9300	C8—H8A	0.9600
C2—C3	1.374 (2)	C8—H8B	0.9600
C2—H2A	0.9300	C8—H8C	0.9600
			
C7—N1—N1^i^	113.69 (14)	C4—C5—C6	119.02 (12)
O2—N2—O1	122.84 (15)	C4—C5—H5A	120.5
O2—N2—C4	118.75 (12)	C6—C5—H5A	120.5
O1—N2—C4	118.38 (14)	C5—C6—C1	118.67 (11)
C2—C1—C6	120.76 (14)	C5—C6—C7	119.49 (11)
C2—C1—H1A	119.6	C1—C6—C7	121.84 (12)
C6—C1—H1A	119.6	N1—C7—C6	115.05 (11)
C3—C2—C1	120.81 (14)	N1—C7—C8	125.53 (12)
C3—C2—H2A	119.6	C6—C7—C8	119.41 (12)
C1—C2—H2A	119.6	C7—C8—H8A	109.5
C2—C3—C4	117.74 (12)	C7—C8—H8B	109.5
C2—C3—H3A	121.1	H8A—C8—H8B	109.5
C4—C3—H3A	121.1	C7—C8—H8C	109.5
C5—C4—C3	122.99 (13)	H8A—C8—H8C	109.5
C5—C4—N2	118.04 (12)	H8B—C8—H8C	109.5
C3—C4—N2	118.94 (12)		
			
C6—C1—C2—C3	1.1 (2)	C4—C5—C6—C1	0.12 (19)
C1—C2—C3—C4	−0.5 (2)	C4—C5—C6—C7	179.49 (11)
C2—C3—C4—C5	−0.3 (2)	C2—C1—C6—C5	−0.9 (2)
C2—C3—C4—N2	177.71 (14)	C2—C1—C6—C7	179.72 (13)
O2—N2—C4—C5	1.6 (2)	N1^i^—N1—C7—C6	−179.90 (13)
O1—N2—C4—C5	179.73 (15)	N1^i^—N1—C7—C8	−0.7 (2)
O2—N2—C4—C3	−176.58 (16)	C5—C6—C7—N1	24.84 (17)
O1—N2—C4—C3	1.6 (2)	C1—C6—C7—N1	−155.80 (13)
C3—C4—C5—C6	0.5 (2)	C5—C6—C7—C8	−154.43 (14)
N2—C4—C5—C6	−177.55 (11)	C1—C6—C7—C8	24.9 (2)

Symmetry code: (i) −*x*, −*y*+1, −*z*.

### Hydrogen-bond geometry (Å, º)

**Table:**

*D*—H···*A*	*D*—H	H···*A*	*D*···*A*	*D*—H···*A*
C3—H3*A*···O1^ii^	0.93	2.57	3.239 (2)	129

Symmetry code: (ii) −*x*+2, *y*+1/2, −*z*+1/2.
